# American Medical Association

**Published:** 1883-07

**Authors:** 


### Society Reports.

### ARTICLE X.

AMERICAN MEDICAL ASSOCIATION. Stated Meeting June 5, 1883.

The meeting was called to order by the Chairman of the Committee of Arrangements, Dr. X. C. Scott, and opened with prayer by the Bishop of the Cleveland diocese, Right Rev. Richard Gilmoure. Dr. Scott then introduced the president of the association, the venerable Dr. J. L. Atlee, of Lancaster, Pa., who assumed charge of the proceedings in a few appropriate words, and introduced Gen'l Ed. S. Myer, of Cleveland, who advanced to the front of the stage and, after pronouncing an eloquent eulogy upon the character and purposes of the association, welcomed them right royally to the homes and hospitality of the citizens of Cleveland, and wished them godspeed upon their labors.

After the address of welcome, the President invited the President and Vice Presidents of the State Medical Society of Ohio, then in session, to seats on the platform, including Dr. H. K. Cushing, (great applause). Dr. Scott explained this action by stating that at the meeting of the Association last year, the State Medical Society of Ohio was invited to hold its Annual Session in Cleveland at the same time, and the privileges of members extended to them for one year only. A specially large committee of arrangements had been appointed with this end in view. The proposed programme for the entire session was then announced, with the arrangements for the separate meetings of the different sections and their places of meeting. The meeting of the association of American Medical Editors was announced at 7:30 in the evening, in Case Hall, previous to the reception in the Euclid Avenue Opera House at 9 p. m. Invitations were extended from a number of prominent citizens proffering the hospitality of their homes on Wednesday and Thursday evenings.

The chairman of the committee of arrangements called the attention of the association to various points of interest about the city. To the parks, the different manufacturing interests, to the rapid growth of Cleveland during the past decade, to the number of children in the schools, public libraries, electric lights and monuments, (the lower part of the city is illuminated by clusters of immense Brush Electric Lamps upon the heads of tall iron masts towering nearly 270 feet high. Euclid avenue, is also illuminated with the Brush light.) Members of associations in Canada were invited to participate in the proceedings. Dr. Scott also announced that every permanent member and delegate was expected to sign a pledge to support the code of ethics of this Association. Protests from various members against leaving out the names of those not having signed the national code of ethics, were read and referred to the Judiciary Committee. The annual address of the President was then read by him. It was unfortunate that the acoustic properties of Case Hall were unequal to the delivery of Dr. Atlee's address to a greater number of the audience than those immediately around him. However, his venerable appearance and dignified bearing, together with the universal respect felt towards him as one of the fathers of the profession, obtained for him the closest attention of his audience to its close.

After the loud applause subsided Dr. X. C. Scott announced the return rates of fare upon all the roads leading out of Cleveland.

Dr. Keller, of Ark., made a motion that the thanks of the association be returned to Dr. Atlee, for his interesting address; and that the paper be referred to the committee on publication. Unanimously carried. Dr. Hedge of Pa., then moved that the members of the Ohio State Medical Society be invited to sit with this association and receive its privileges for one year. Carried. The president then announced that each state delegation, at the close of this session, meet and appoint one of its members as its representative, in the election of officers of the association for 1884.

Dr. Robinson, of Nashville, then asked for the report of the committee on credentials. After some delay the President announced that the committee were not ready to report. Dr. Billings, U. S. A., presented a communication from Dr. Mahomed, calling the attention of the association to certain action last year of the British Medical Association, on the subject of atmospheric conditions and their reference to disease, and moved they be referred to the appropriate committe on Hygiene. Carried.

Papers from Dr. Stewart on "A New Operation for Ranula," and on "Warren's New Cure for Hernia," were referred to their appropriate committees. A communication was read by the secretary from Dr. Dwight W. Joy, asking for a re-hearing of the evidence in his case, and referred to the Judicial Committee.

Dr. Didama, of N. Y., presented a communication from a New York member unable to attend by reason of exclusion of his State Society, as to the propriety of petitioning Congress to provide stations for additional meteorological observations in localities said to be suitable for the treatment of pulmonary diseases.

The Secretary then read the list of names of members registered. Some confusion arising, Dr. S. W. Gross, of Pa., suggested that, as many members were leaving the room, further reading be dispensed with. Dr. Toner, of Washington, explained that the reading was a formal decision as to membership. Discussion arose on the stage as to the propriety of each member answering to his name when called. The president decided however, that as all the members had registered the same morning it would not be necessary to answer to names.

After the reading of the names the Association adjourned to 9:30, Wednesday.

On the stage were the Vice Presidents of the assocation, Dr. H. S. Orme, of Cal., Eugene Grissom, N. C., J. A. Octerlony, of Ky., A. J. Stone, Minn. Immediately after adjournment the state delegations each drew apart and selected representatives to nominate officers for 1884. The Pensylvania delegation met on the stage near tbe reporters table and nominated the venerable Dr. S. D. Gross, as their representative, instructing him to cast the vote of Pennsylvania for Austin Flint, Sr., for president for 1884, an amendment allowing him to use his discretion being voted down.

FIRST DAY.

*The Section on Practical Medicine* met in Y. M. C. A. Chapel, Dr. J. H. Hollister, of Ill., in the chair, Dr. J. G. Lee, Secretary.

Dr. R. D. Murray, presented a paper on yellow fever, which was read by T. W. Miller, of Ill. Quite free discussion followed by Drs. Campbell, of Ga., Palmer, of Ohio. Beach, of Mich., Bell of N. Y., Franklin, of Ohio, and Dr. J. B. Hamilton, of the Marine Hospital service. Paper referred to the committe on publication.

Dr. Wm. M. Beach, of Ohio, then read a paper on "Milk Sickness," which was discussed to some extent by Dr. Palmer, of Mich.

Section then adjourned to 2.30, Wednesday.

*Section on Gynæcology and Obstetrics.*—A full attendance present. Dr. J. K. Bartlett, of Wisconsin, in the chair. Dr. G. H. Moses, Secretary. First paper, by Dr. W. H. Byford, of Illinois, on "Chronic Intra-Pelvic Inflammation," read by the Secretary, Dr. Byford being unable to appear in person.

Dr. Byford's object in writing the paper was to draw the attention of the profession to the danger of converting a chronic pelvic inflammation into a disastrous acute form, and gave the following summary of suggestions:

1st. The sometimes terrible effects of examinations or operations in the pelvis do not often take place when there is not a perceptible predisposing inflammation.

2d. The inflammation may be so slight as to be easily overlooked.

3d. It may be an original condition; the sequence of an attack long gone by; or it may be the product of some immediately previous examination or operation, the effects of which have not subsided.

4th. To avoid the dangers of acute inflammation, we should, in making a first examination for pelvic disease, conduct it in such a way as not to give the patient much pain, and when she complains of pain, should desist, even at the sacrifice of complete diagnosis.

5th. Complaints of much tenderness to the touch or the use of instruments, especially in primiparous women, are sufficiently diagnostic of inflammation upon which to base treatment for that condition.

6th. If, with such tenderness, a thorough examination or an operation is imperative, it should be done under profound anæsthesia. There is no question but much less danger of ill effects is incurred in making examinations or operations upon susceptible subjects under the free use of anæsthetics.

7th. Examinations or operations should not be repeated till the effects of the first have entirely passed off.

8th. As chronic parametritis is a frequent complication of most of the morbid conditions of the uterus, it should always be inspected, and its diagnosis be carefully considered in all cases of metritis.

9th. When chronic parametritis is present, it should be the chief, if not exclusive object of treatment until removed.

10th. It is not safe to use the uterine stem when there is perimetric inflammation.

11th. It is especially dangerous to explore a displaced uterus when it is bound down by inflammatory adhesion, by any means which will overcome its fixedness by force.

12th. All local treatment of the uterus must be conducted with the greatest care in all cases where the complication is present.

The second paper was one by Dr. H. G. Landis, of Ohio, subject, "Post-Partum Polypoid Tumors," with two cases.

Third paper by Dr. H. O. Marcy, of Boston, Mass., on "Restoration of the Perineum by a New Method."

This new method consists in the use of German-silver wire for the sutures. This possesses elasticity sufficient to give lateral support when the ends are bent together, forming external support to the refreshened tissues.

The fourth paper in this section was one by Dr. R. S. Sutton, of Pennsylvania, entitled "Enterotomy as a Complication in Ovariotomy or Oöphorectomy," he having removed four inches of the small intestine a few months since successfully; the first case in this country, the only other one on record being by Billroth, of Vienna.

Section adjourned to 2:30 Wednesday.

*The Section on State Medicine* met in the U. S. Court-room, in the Post-office building, a small attendance present, owing to conflicting announcements of the meeting at different places. The only paper presented was by Dr. A. L. Gihon, U. S. A., on "Medical Education the Fundamental Fact in Medical Ethics." Great interest was manifested in the paper, which was an extremely able production, the writer's position as member of an Examining Board for admission to the medical service of the United States Navy qualifying him admirably for observation of the educational attainments of this country. It is to be regretted we cannot give the paper in full.

*Surgical Section, First Day*, met in Case Hall, at 2:30 P. M., Dr. W. F. Peck, of Iowa, chairman; Dr. Paul F. Eve, of Tennessee, secretary. Attendance quite large; many ladies present.

First paper, R. A. Vance, on "A Radical Cure for Hernia by a New Method," which consists, in oblique inguinal hernia, of uniting the pillars of the ring by means of a deep suture passed subcutaneously with a peculiar semicircular needle.

Dr. Dudley P. Allen, of Cleveland, read a paper on "Comparison of Antiseptic and Non-Antiseptic Methods." In conclusion, the essayist dwelt upon the fact that different methods are of different application, and that while the spray might be most desirable in opening joints and in the atmosphere of hospitals, with hygienic surroundings, flooding might be equally efficient in certain other wounds, and that some prominent antiseptic such as iodoform, would be most appropriate where other antiseptics are inapplicable, as in removal of the tongue. Then, though there are certain dangers in the use of antiseptics, these are more than equalled by the dangers attendant upon their omission, especially in large hospitals, and that dangers of poisoning are certainly decreasing, as the use of antiseptics is better understood; and, further, that investigation may develop a method of securing antiseptic results less onerous and devoid of the disadvantages which now surround them. In conclusion, he expressed his belief that the various antiseptic methods secured far better results than any other method.

In the discussion which followed, Dr. Mortimer, of Massachusetts, expressed the belief that Listerism would soon be a thing of the past, which was strongly taken exception to by Dr. Nancrede, of Philadelphia. Several others participated in the discussion.

The next paper, on the "Value of Early and Late Operations in Morbid Growths, Especially Malignant," was read by Dr. Samuel D. Gross, of Philadelphia, whose appearance was greeted with applause, and the paper received the profound attention of the audience throughout.

The paper strongly dwelt upon the importance of early operation in morbid growths, especially malignant, as soon as the diagnosis is unmistakable. The author sketched the mode of development of most morbid growths, and the points of special diagnosis indicating the operation for their removal, dwelt strongly upon the necessity of thorough knowledge of pathological anatomy, a branch of science, little cultivated in any of our schools, and almost totally neglected in most. It is not surprising that the art of diagnosis should be so little understood by the generality of practitioners, and so many errors committed in the examination of morbid growths. If there is any one thing in the organization of our medical colleges more culpable; I had almost said, more criminal, than any other, it is the exclusion from their curriculums of the study of pathological anatomy. Just in proportion as our knowledge of morbid structure is positive, accurate and comprehensive, will be the probability that we shall become skilled diagnosticians, and conversely. Hence, so long as this state of things exists, we shall look in vain for any marked improvement in this direction, and what is true in this respect is true alike of city and country practitioners, standing as they do upon the same unfortunate platform.

Dr. H. A. Martin, of Massachusetts, then followed with a paper upon the "Treatment of Synovial Diseases by a New Method," which consists of aspirating the sac and applying the rubber bandage to the joint.

Section then adjourned.

*Section on Diseases of Children* met at 2:30, in the City Council chamber. Neither chairman or secretary present. Dr. Chas. W. Earle, of Chicago, called to the chair, and Dr. E. L. Boothby appointed secretary.

None of the readers of papers on the day's programme having appeared, Dr. Earle read a short paper on "Cephalohæmatoma in the New-Born;" the most important question connected with which, the writer said, was diagnosis, and then gave four points for differentiating, namely, from caput succedaneum, which is œdematous, and does not fluctuate, being only a difficulty of the scalp and cellular tissue; congenital encephalocele never occurs on the cranial bones; a vascular tumor has sometimes the same boggy feeling noticed in caput succedaneum, but it has not the bony ridge surrounding cephalo-hæmatoma; cranial tabes is the name given to the softened cranial bones in children. As to treatment, the author advocated judicious non-interference. An extended discussion was indulged in upon the subject by Drs. Harris, Reed and Earle.

Adjourned.

*Section on Dental and Aural Surgery* met at 2:30, at the rooms of the Vocal Society. Dr. D. H. Goodwillie, of New York, in the chair, Dr. T. W. Brophy, of Ill., Sec'y.

Papers on the "Relations of Teeth to Other Organs," and "Amaurosis Dependent on Dental Irritation," were not read, as the authors were absent. The remaining one on "Erosion of the Teeth," was read by Dr. John H. Marshall. After this paper closed Drs. Henry, Noyes, Nichols and Benton, of New York, made emphatic objection to Dr. Goodwillie presiding over the meeting—having been informed that Dr. G. had registered as a delegate from the New York society, which was not allowed a delegation to the convention, under a protest from the judicial board. Dr. Goodwillie then resigned the chair to the secretary and Dr. Williams was elected temporary chairman. Dr. Marshall's paper was then discussed and the section adjourned.

*Section on Ophthalmology, Laryngology and Otology* met in the Board of Education rooms at 2:30 P. M. The chairman, Dr. A. W. Calhoun not present, Dr. J. J. Chisholm, of Maryland, nominated and elected. Dr. Carl Seiler, of Philadelphia, secretary.

First paper by Lawrence Turnbull, of Pa., on "Paralysis of the Facial Nerve in connection with Diseases of the Ear." Little discussion followed. Second paper not read. Third, a fine paper by Dr. W. B. Jarvis, of N. Y., on "Tonsillotomy by Ecrasement." The author advocated the use of the snare in the cases of hard hypertrophied tonsils (scirrhous tonsils) to avoid hæmorrhage. Paper was largely discussed. Most of the members present claimed that the hemorrhage was met in but comparatively few cases. Fourth paper by Dr. Carl Seiler, of Philadelphia, on the "Action of Nitrate of Silver on the Mucous Membrane of the Throat," read. The substance of the paper was that nitrate of silver is not a caustic in any strength, but that the slough often seen in such cases is due to localized inflammation caused by granules of oxide of silver deposited in the subepithelial tissues. Paper also largely discussed. The 5th and 6th papers read by title only. Authors not present. They were "Tumors of the Post Nasal Space," by Dr. E. Fletcher Ingals, Ill., and "Myringitis," by Dr. C. Williams, of Minn. Section adjourned.

### WEDNESDAY, JUNE 6—SECOND DAY.

The Association was called to order at 9:30 A. M., by the President, and prayer offered by Rev. Charles S. Pomeroy, D.D.

The Secretary then read the names of the members who had been delegated by their respective States to nominate officers for the ensuing year, as follows:

W. O. Baldwin, Ala.; D. A. Linthicum, W. T. McNutt, Cal.; T. M. Hill, Conn.; H. K. Steele, Colo.; W. Marshall, Del.; D. C. Patterson, D. C.; E. Foster, Ga.; C. T. Parkes, Ill.; H. J. Wood, Ind.; W. S. Robertson, Iowa; L. S. McMurtry, Ky.; J. W. Dupree, La.; C. A. Savory, Mass.; J. J. Chisholm, Md.; B. H. Miller, Minn.; F. K. Owen, Mich.; E. H. Gregory, Mo.; A. J. Fuller, Me.; V. H. Coffman, Neb.; D. A. Watson, N. J.; E. Grissom, N. C.; H. D. Didama, N. Y.; W. M. Beach, O.; S. D. Gross, Pa.; A. Ballou, R. I.; R. A. Kinloch, S. C.; D. J. Roberts, Tenn.; H. C. Ghent, Tex.; A. Harris, Va.; J.

M. Lazalli, W. Va.- S. C. Johnson, Wis.; J. R. Smith, U. S. A.; T. U. Miller, U. S. Marine Hospital Service; A. L. Gihon, U. S. N. W. A. Tipton, N. M.; A. B. VanNelson, Dakota.

At the close of the reading, Dr. Pratt, of Michigan, called attention to the amendment to the constitution which he offered at the last meeting, to the effect that none but members present should be eligible to the offices of President, Vice-Presidents, Secretaries and Chairmen of sections, the Assistant Secretary, and members of the Judicial Council.

On motion, the amendment was taken from the table and adopted.

Dr. S. D. Gross presented a paper, signed by Dr. O. W. Holmes, Dr. Austin Flint and himself, recommending the memorializing of Congress on the subject of an appropriation for the National Medical Museum and Library.

Dr. H. A. Johnson then presented the following resolution:

*First*, That the American Medical Association respectfully urges upon Congress the importance of at once providing a commodious fire-proof building to contain the Army Medical Museum and Library.

*Second*, That the annual appropriation for the library should be sufficient to enable it to obtain all new medical publications of all countries as soon as they appear, and also to complete its collection of medical books heretofore published, and that, for that purpose, the sum of $10,000 is considered a reasonable and proper annual appropriation, and Congress is requested to grant that sum, in addition to the amount required for the Medical Museum.

*Third*, That it is of the greatest importance that the index and catalogue of this library now in course of publication should be prepared as rapidly as possible for the press, and Congress is urged to make the necessary appropriation for the purpose.

*Fourth*, That a special committee of five be appointed, of which the President shall be *ex officio* chairman, to present this matter to Congress, and call the attention of State and local societies to the importance of furnishing Senators and members of Congress with information as to the value of the museum and library, and the estimation placed upon them by the medical profession of the United States.

These resolutions were adopted.

An invitation was then read extending an invitation to the Association to visit River Bank, six miles out on the lake shore, the country seat of Mr. D. P. Eels, a train being provided free of charge.

Dr. N. S. Davis read the report of the Board of Trustees appointed at the last meeting to conduct the process of establishing a journal, to be known as *The Journal of the American Medical Association*, in which the history of their proceedings was fully set forth. Nine members had constituted this board, of which Dr. N. S. Davis had been chosen President, and Dr. J. H. Packard, Secretary. The plan decided upon was to issue a journal containing thirty-two double-column pages of reading matter, suitably divided into departments of editorial, devoted to discussion of topics likely to promote the interests of the profession; an editorial department devoted to summary of the progress in various departments of medical science and practice; a department of correspondence, and for general items and intelligence.

Forty thousand circulars had been issud to members of the profession on the subject, each enclosed with a blank for reply. 2,150 answers had been received, of which 2,100 were unqualified expressions of approval of the plan. It was calculated that the aggregate subscription to begin with would be 2,500, which would produce an income of $12,500 yearly. The advertisement patronage, under fair business management, would be probably not less than $5,000 annually, which would yield an income of $17,500 per year. The expense of printing 3,500 copies weekly was estimated at $8,000 per year, leaving $9,500 for salaries and other expenses. The salaries were estimated at not more than $6,000, leaving $3,500 for other expenses, such as scientific investigation in any direction determined upon by the Association. The Board had determined to recommend the publication of the journal—the place of publication to be in Chicago, the printing to be awarded to A. G. Newell, of Chicago. In conclusion, the reader introduced the following resolutions:

*First*, That the report of the Board of Trustees be accepted, and the recommendations contained therein be adopted with reference to establishing the journal.

*Second*, That the Board of Trustees be instructed to proceed with the publication of the journal at as early a date as possible, to take the place of the annual volume of "Transactions;" also to the effect that all orders upon the treasury for the payment of money be indorsed by the president of the Board of Trustees.

Dr. Brodie moved the adoption of the resolutions. Dr. Wylie moved as an amendment to the motion, that the report be printed and laid over till next day for discussion. The amendment was lost, and the motion carried by a large majority.

Dr. McMurtry, of Kentucky, of the Board of Trustees, announced that he had been instructed by the board to announce that it had unanimously selected Dr. N. S. Davis, of Illinois, as its editor in chief.

Dr. Davis then made an address, expressed his feeling of inability to comply with the requirements of the position, and stated some of the difficulties to be met with, warning the members not to expect too much of the new undertaking, or compare it too strictly with the *British Medical Journal*, which had been the work of years. He hoped, also, to be able to issue the first number in July next. He then resigned his membership of the Board of Trustees.

Dr. Cohen, of Philadelphia, moved that the Board of Trustees be instructed, in addition to the journal, to print annually a thin octavo volume containing the minutes of the Association. Discussion followed by Drs. Hubbard, Quimby and Byrd. Dr. Richardson, of Louisiana, moved to refer it to the Board of Trustees. Carried.

Dr. Busch, of Delaware, moved a vote of thanks to Dr. N. S. Davis for his long service to the Association. Carried.

There being four vacancies in the Board of Trustees, a committee were appointed to nominate the proper quota of members. Drs. Richardson, of Louisiana, Brodie, of Michigan, Hibbard, of Indiana, and X. C. Scott, of Ohio, constituted the committee.

Dr. Davis announced, in answer to a question, that the Judicial Council assumed all responsibility in placing the pledge to support the code of ethics of the American Medical Association on the blanks to be signed by delegates before registering.

Dr. Palmer, of Michigan, asked if the present condition of the code was meant especially, or whether those who signed were expected to sustain all subsequent alterations as well?

Dr. Davis said, in reply, that any changes made by the Association would be considered binding on the members who signed the code. He explained also, that the custom of signing the code originated in the early days of the Association, when the members, as initiated, signed their names to the Constitution and By-Laws, but as the Association increased in numbers, the custom being clumsy and inconvenient, was allowed to lapse. Last year the question came up, and demanded a definite answer, What is the Code? A card was then provided for the signature of the loyal members.

Dr. Scott announced several additional protests against registration of certain members, which were referred to the Judicial Council.

The address of the Chairman of the Section of Practical Medicine was given by Dr. J. Hollister, of Illinois, on "The Progress of Medicine during the Past Year." The speaker dwelt largely on the results of microscopical investigation; the importance of the investigations of Koch, Bizzozero, Norris and others; made extended reference to the micro-organisms of leprosy, of gonorrhœa, the vaccine pustule, etc., and said that the question which concerns us most is not whether we can destroy bacteria, but whether they have not a greater vitality than the tissues of the human body, and whether, in the germicidal warfare, the human organism will not first succumb. In materia medica new remedies had been brought forward, but none seemed sufficiently important to attract special attention in the year's reports.

In conclusion, the speaker urged upon the attention of medical men the propriety of establishing a Medical Bureau, composed of say ten members, each to be appointed by the President, upon the recommendation of a nominating board consisting of one member carefully chosen by the profession in each State, the army and navy to be also represented. Let the members of this bureau be subject to removal only for cause, and each receive a salary of not less than ten thousand dollars annually, to be paid by fees from those who are applicants for the degree of Doctor of Medicine. Let the laws of the separate States be so modified that the power of conferring degrees shall rest solely with this body. Let students graduated by this National Medical Bureau receive an honorary distinctive title, say that of National Fellow of Medicine. To such a movement, the colleges could offer none but selfish objections.

In such a plan as this there would be found the possibilities of a medical culture of the profession such as the world has not yet seen.

At the close of this paper, the Rocky Mountain Medical Association was announced to meet at the Weddell House in the evening. This consists of those surviving members and their wives who attended the last meeting of the Association in San Francisco.

The Committee on Vacancies in the Board of Trustees for the new journal reported the selection of Drs. G. O. Hooper, of Arkausas, Alonzo Garcelon, of Maine, and L. S. McMurtry, of Kentucky, to fill out the expired terms of office; Dr. J. H. Hollister of Illinois, to fill the place resigned by Dr. Davis.

The next proceeding being the address of the chairman of the Section in Obstetrics and Diseases of Women, by Dr. J. K. Bartlett, of Wisconsin, the President announced that, as Dr. Bartlett's voice was impaired, the paper would be read by Dr. Nicholas Senn, who then read the address, which presented a *resumé* of the progress of gynecological and obstetrical surgery.

Of Emmet's operation he said that prolonged observation had shown that undue influence had been attributed to the lesion, and experience has proven that the relief claimed to follow the operation is not uniformly attained.

Battey's operation received due consideration, and the conclusion reached that there are still reasonable doubts as to its efficacy in epilepsy or mania dependent, on Ovaritis.

Tait's operation was considered, and reference made at some length to the treatment of extra uterine pregnancy by electricity. As to the use of transfusion in cases of post-partem hemorrhage, confidence in it as a valuable therapeutic measure is on the wane. As to the obstetric forceps, the author spoke in terms of strong reprobation of the motive for their use, of hurrying the abor to secure time for a second engagement. Goodell's memorable observation forcibly quoted: "To tell you the truth, such grave lesions to the mother and to the child also, are so constantly brought to my mind, that I am disposed to accept Baudeloque's dictum that, take it, all in all, the forceps have been more injurious than beneficial to society.

The judicious administration of ergot in the second stage of labor, regarding it as a valuable resource in cases of insufficient contraction, with no pelvic obstacle.

Anæsthetics in labor were discussed with the conclusion that if an anæsthetic ever produced disastrous results, it was due to the impurity of the agent, or want of proper discretion in the operator.

The subject of antiseptics received conservative treatment at length; and in conclusion, the speaker dwelt upon the absolute necessity of careful constitutional treatment in pelvic disease, claiming it had not received the attention due to it.

In conclusion, he remarked that specialism, when exclusively practiced, is apt to produce narrowness of view, and that the time when the present brilliant triumphs of the surgical gynæcologist will grow pale before the achievements of his medical co-workers.

Dr. Toner, of Washington, then presented his report on Necrology, which was referred to the Publication Committee.

The Association then adjourned.

*Section on Practical Medicine* met at the Euclid Avenue Opera House, Dr. J. H. Hollister, of Illinois, in the chair, Dr. J. G. Lee, secretary.

The first paper, by Thos. W. Rennolds, of Michigan, was on the subject of "The Alimentary Canal in Bronchitis and Phthisis."

The speaker said the abnormal condition of the portal and lacteal systems was often the predisposing cause of both acute and chronic affections in all parts of the respiratory tract. Acute tracheo-bronchitis was often caused by excess in the dietary elimination, with proportionally incomplete waste.

In view of this fact, in such cases the treatment should be prompt evacuation of the bowels, and restriction of the diet to a light liquid form.

He deprecated the use of ordinary cough mixtures to the exclusion of this more rational treatment. Morphine, quinine, aconite and veratrum viride were usually the more appropriate remedies in the first stage, but did not equal this treatment without drugs to which he referred.

Derangement of the digestive system was still more causative of chronic bronchitis, and the treatment should have reference to this fact; where purulent, quinine was the best remedy in connection with the management pertaining to proper elimination.

Clothing should be sufficient, but not oppressive. A common mistake was wearing too much on the chest. He had several times seen striking improvement in expectoration follow the removal of two or three extra undershirts and a chamois leather lung protector.

Physical exercise involving free expansion of the lungs restored wonderfully their normal elasticity after an attack. It dissipated thickening and adhesions, just as continued free motion dissipated the thickening and adhesions around a recently inflamed joint.

Catarrhal fibroid phthisis was most frequently the result of neglected chronic bronchitis and should be treated in much the same way, not by Cod-liver oil alone or any other supposed specific, especially if they interfered with digestion ; but the patient should cultivate out-door life, with plenty of physical exercise and wholesome mental occupation. Any region free from malaria, or unwholesome emanations, with a temperature permitting constant out-door life would answer for a resort.

At the close of the paper, Dr. W. T. Belfield, of Chicago, gave some illustrations of the micro-organisms of disease, with an oxy-hydrogen apparatus and solar microscope kindly furnished by Dr. McIntosh, of Chicago.

Dr. Belfield accompanied the exhibition of the slides with a running commentary upon the peculiar organism displayed.

At the close of the lecture Dr. Austin Flint, Jr., made some interesting extempore remarks ; also Dr. Palmer, of Michigan, who offered Milk Sickness as a subject for microscopical investigation to histologists.

A paper followed by Dr. John V. Shoemaker, of Pennsylvania, on Mechanical Remedies in Skin Diseases. These are, according to the essayist, massage, compression, blood-letting, incision, excision, enucleation, scooping, scraping, setons, cauterization; remedial measures which have been in vogue almost from time immemorial. The speaker exhibited his new dermatome and other appliances.

The session closed with a paper by Dr. L. B. Tuckerman on a new method of obtaining pancreatic juice, which was practically illustrated upon the animal. The process proved one of the most interesting events of the afternoon.

*Section on Surgery and Anatomy—Second Day.*—The chairman, Dr. Peck, called the section to order at 2 P. M., half an hour earlier than usual.

The chairman appointed a sub-committee on papers, consisting of Drs. McMurtry, of Kentucky, Moon, of New York, and Parkes, of Illinois, after which Dr. Robert Newman, of New York, read a paper on The Surgical Use of Electrolysis in Stricture of the Urethra.

On motion, the section agreed to hear the paper by Dr. J. R. Taylor, of New York, which was on the programme for the next day, on account of his compelled departure this evening.

The paper was on The Treatment of Fractures of the Long Bones, and was well illustrated by engravings and apparatus applied to the subject. The uncomfortable perineal band in fracture of the femur he has transformed into a comfortable sort of saddle which distributes the pressure of counter extension over the whole perineum. This saddle is made fast by straps to the headboard of the bed, while extension is made by a screw in the footboard. In the adjustment of fractured ribs the Doctor brings the broken ends into place by raising the arms over the head, maintaining apposition by means of adhesive plaster closely applied. Of his treatment of fractured clavicle the author gave an illustration on the subject: how the traction is made mainly upon the body of the sca ula, securing thereby perfect immobility. He contended strongly for simplicity and readiness in surgical apparatus and procedure.

Dr. Henry O. Marcy, of Boston, then read a paper on "The Comparative Value of Antiseptics." This paper consisted of a detailed series of experiments with a full list of the known antiseptics, with rather surprising results in some instances.

Dr. L. H. Sayre, of New York, then read a paper entitled "Amputation below the Knee Joint in Preference to Brisement Forcé or Resection in *Certain Cases* of Deformity with Anchylosis." Illustrated by two cases; the first, of a lady who suffered amputation and walked upon an artificial limb in six weeks after operation, the second case an amputation performed six weeks since, in which the stump has thoroughly healed.

The next paper, "Report of a Case of Re-amputation at the Hip Joint; Secondary Hæmorrhage on Sixth Day; Ligature of the Primitive Iliac Artery," was received and referred without reading to the committee on publication.

Dr. E. M. Moon then read a very able paper on "The Treatment of Unreduced Dislocations of the Ulna in connection with Colle's Fracture." This paper excited prolonged discussion.

Another interesting paper was read by Dr. V. H. Coffman, of Omaha, Neb., entitled "Treatment of Tender Spines by Subcutaneous Incision." Close attention was given during the reading, and at its close many questions were asked the author as to his methods.

Session then adjourned.

*Section on State Medicine.*—Dr. Foster Pratt, of Michigan, in the chair.

Dr. H. A. Johnson explained the results accomplished by the Illinois State Board of Health. Since 1877, the date of its organization, about three thousand quacks, itinerants and nondescripts, who combined other callings with that of doctor, have been driven out of the State. The discussion following this paper occupied the time till adjournment, except a resolution introduced by Dr. Pratt, recognizing the labor and life work of the late Dr. William Farr, of England, in the organization, classification and compilation of vital statistics as an enduring monument to his ability and learning as a physician as the real incentive to and the foundation of our own sanitary work, and as a perpetual blessing to present and future generations of our universal humanity; entitling his name and fame to stand with that of other great men whose genius and labors have resulted in beneficent revolutions of the medical, surgical and sanitary thought and activities of the civilized world.

*Section on Gynæcology and Obstetrics.*—Meeting at Frohsinn Hall at 2 :30 p. m. Dr. Bartlett in the chair. Dr. J. T. Jelks, of Arkansas, secretary.

The first and second papers not appearing, Dr. John Morris, Maryland, read his paper on "What Means Can be Judiciously Used to Shorten the Time and Lessen the Hours of Labor." The author described lingering labor, dividing it into three stages: First, when the head remains high up in the pelvis; second, when it has well descended into the pelvis, but the parts are tense and undilatable; third, when the head impinges upon the perinæum. He explained the procedures necessary at each of these stages, and when to use the last resort, the forceps. The paper was discussed mainly by Drs. McClure, of Pennsylvania; Reamy, of Ohio; Smart, of Michigan; Gordon, of Maine; Landis, of Ohio; Humiston, of Massachusetts; Robinson, of Pennsylvania, and others.

Dr. E. C. Dudley, of Illinois, then read his able paper on "The Immediate Application of Sutures in Puerperal Laceration of the Cervix and Perinæum." Paper discussed by Drs. Walker, of Kentucky; Jenks, of Illinois; Morris, of Baltimore; Maughs, of St. Louis; Ulrich, of Pennsylvania, and Parsons, of Michigan.

The closing paper of the session was one volunteered by W. N. Taylor on The History of Laparo-Elytrotomy, with a case detailed.

The section then adjourned.

*Section on Diseases of Children*—Met in the Council Chamber at 2:30 p. m., Dr. R. F. Blount in the chair. Dr. E. L. Boothby elected Secretary for the remainder of the session.

Dr. A. Y. P. Garnett, of Washington, read a paper on "Epidemic Jaundice Among Children," detailing an epidemic of this nature which had come under his care, for which no apparent cause could be assigned. Some discussion followed.

Dr. Alexander Harris, of Virginia, then read a paper on the "Unity of Diphtheritic and Membranous Croup." The doctor claimed that the diseases are identical; that the difference in the degree of adhesiveness in the membrane is due only to the structure of the parts on which they exude. A warm and interesting discussion followed from nearly all the members of the section, the discussion being about equal between the unicists and dualists.

Dr. W. N. Myer, of Fort Wayne, Ind., presented a paper on "Surgical Treatment of Purulent Pleuritic Effusions." The author detailed a case of Empyema, giving his opinion that free incision should be employed in Empyema in childhood, and aspiration only in serous effusion.

Dr. Chas. W. Earle, of Illinois, then read an able and interesting paper on "A Plea for Pleasant Medication for Children and a more thorough Study of Infantile Therapeutics." He advised the profession to spend less time on the curve of a forceps or the shape of a bacteria, or the riding of gynæcological hobbies, and devote more attention to the making of palatable medicines for children. He suggested many ingenious methods of disguising and rendering palatable bitter and nauseous medicines. Some discussion followed.

Section then adjourned.

*Section on Dental and Aural Surgery.*—Section met in the Vocal Society rooms. Dr. Williams in the chair. None of the papers on the programme were present.

Dr. John Marshall reported a case of Caries of the Maxillary Bone. Considerable discussion followed which corroborated the experience of the speaker.

Dr. Talbot reported a case of Septicæmia from an alveolar abscess. After general discussion the section adjourned.

*Section on Ophthalmology, Otology, etc.*—Met at Board of Education, 2:30 P. M., second day. Dr. J. J. Chisholm, in the chair. Dr. Carl Seiler, Secretary.

First paper not on hand.

Second paper: "A Case Illustrating the Segmental Feature of Glaucoma." The drift of this paper was that Glaucoma would show itself occasionally in segmental distribution. The author discussed at length the pathology of Intra ocular Inflammation. Paper largely discussed.

Paper No. 3 on the programme not read. No. 4 also, but read by title.

Paper No. 5 by Dr. Lawrence Turnbull, of Pennsylvania, on "Tinnitus Aurium and the Deafness which Accompanies Bright's Disease." The paper asserted that there are certain aural symptoms peculiar to Bright's Disease. In the discussion following it was maintained those symptoms are not pathognomonic of Bright's Disease.

Paper No. 6, "Questions on the Ætiology of Some Forms of Lenticular Opacity," by Dr. J. L. Thompson, of Indiana, consisted of a collection of cases of peculiar opacity occurring in the lower periphery of the lens, this opacity sudden in occurrence, and lasting for years without change. Considerable discussion followed, the substance of which was that similar opacities had been due to various causes.

Section then adjourned.

### THIRD DAY—JUNE 7.

The Association called to order by the President at 9:30 A. M., and prayer offered by Rev. Dr. N. S. Rulison. The President announced that he had already appointed delegates to the International Medical Congress at Amsterdam, and was now ready to appoint as delegates any members who wished to attend.

Dr. Keller, of Arkansas, called up his amendment to the Constitution offered last year, which provided that the time of the annual meeting be left to the Committee on Nominations, and not limited to the first Tuesday in June. The amendment was adopted.

Dr. Batchelor offered a resolution that the President appoint a committee, consisting of one or more members from each State, to influence legislation on the subject of the sale of intoxicants; adopted.

Dr. Foster Pratt offered the resolution which had been adopted in the Section on State Medicine the day before, in reference to the death of Dr. Wm. Farr; adopted.

Dr. Walter Hay, of Illinois, made a motion that a section on Psychological Medicine be established; laid over one year under the rules.

Dr. S. D. Gross offered a resolution in recognition of the necessity of trained nurses, that the Association recommends that training schools for nurses be established in every county of each State, instruction to be given gratuitously, or at rates which would not exclude the poor from their benefits; carried.

The report of the standing committee on Atmospheric Conditions and their relations to Disease, was made by the Chairman, Dr. N. S. Davis. He stated that a series of observations had been carried on at parallel points throughout the United States of the daily condition of the atmosphere, quantity of ozone, and presence of organic matter in the air. Interesting reports had thus been collected from different localities on the subject of endemic diseases. As to the prevalance of children's diseases of pneumonia, the methods of observation given in detail. The report was accompanied by extensive tables, and much credit given Professor Long as the collaborator, of the amount of organic matter daily present in the atmosphere during the entire past year for the first time in this country or any other. The report closed with an expression of thanks to Gen. Hazen, of the United States Signal Service, for uniform courtesy and favors extended, and a request for a continuance of the same. It was mentioned incidentally that not a physician in New York City could be secured to co-operate with the committee in making observations. The report was adopted.

Dr. Didama then called up the resolution presented by him on the first day—providing for a committee of five to petition Congress to establish observations in localities, said to be favorable to the treatment of pulmonary diseases, for the taking and compilation of climatic statistics on this subject. Referred to Committee on Atmospheric Conditions.

Dr. Reed, of Iowa, offered a resolution of condolence to the family of the late Dr. J. B. Hubbard, of Ashtabula, which was carried.

Dr. Davis moved that Dr. Didama be added to the Committee on Climatology.

The names of the delegates abroad were then read as follows: G. J. Engleman, St. Louis; N. M. Finley, Altoona, Pa.; Walter L. Zigler, Lancaster, Pa.; M. N. Alto, Armstrong County, Pa.;

R. B. Cole, San Francisco; Jos. H. Warren, Boston; C. N. Klein, Hamilton, O.; W. M. Lawlor, San Francisco; Henry Martin, Boston; J. C. Hutchinson, Brooklyn; A. M. Hawes, Detroit; Ed. Borck, St. Louis; T. F. Prewitt, St. Louis; E. P. Allen, Pa.; H. McCall, Mich.; I. M. Quimby, N. J.; S. T. Gordon, Maine.

Dr. Pollak, of St. Louis, offered a resolution on behalf of the St. Louis Medical Society, suggesting that, whereas many of the present provisions of the Code of Ethics are obsolete and that early revision is necessary, and no society except the American Medical Association has power to alter the present Code, but simply to ask for its revision; therefore, that the American Medical Association be requested to appoint a committee of one from each State for the purpose of taking into consideration the propriety of revision of the Code of Ethics of the American Medical Association and report thereon at the meeting of 1884.

It was moved and seconded by numbers of members in every part of the hall, that this resolution be laid on the table, and carried with half a dozen negatives and great applause.

Dr. Brodie, of Michigan, offered a resolution that all papers before being read should receive the approval of the Secretary of the Section in which it belonged.

A member made the invidious remark that many papers presented are not worth the paper they are written on. Resolution laid on the table.

Dr. M. L. Nardyz was received as a member by invitation on motion by Dr. Davis.

The address in Surgery was then delivered by Dr. W. F. Peck, of Iowa. It consisted of a review of the progress in the Surgical Science of the world during the past year made interesting comparison of the various shades of bacterian theory in etiology of disease, and concluded that practical surgery has not thus far been benefited by Koch's views—and that the condition of the problem of the management of wounds and other pathological processes by means of the so-called antiseptic methods suggests a move in the direction of greater confidence in the details of operative procedure and scrutinizing attention in extreme cleanliness in the minutial of practice. Of the progress in invention of instruments the author mentioned the compound ratchet splint offered by Dr. Stillman, of New York, of the various modifications of the Swan electric lamp for exploring the cavities of the body, mentioned the satisfactory progress in abdominal pathology, discussed Gastrotomy, Laparotomy, and the overlooked frequency of pathological conditions in the ilio-cæcal region; gave a case in detail of exploration of the abdominal cavity by incision, and the discovery of an obstruction of the bowels by a singular abnormal position of the vermiform apendix, which would have eventuated in death. The author dilated upon the importance of boldly opening the abdomen before the case becomes desperate. It proved an extremely interesting paper and received close attention to its close.

The President then introduced Dr. Foster Pratt, of Michigan who read the paper on the progress of sanitary science during the past year. Said he had no discoveries to offer, but could report progress, great especially during the past fifteen years. Municipalities are improving in methods of sanitary precaution. Attention has been called to the heating and lighting of our houses and other buildings. The causes of disease we find largely to depend upon fixed physical laws, and by the discovery of these we are able to prevent disease. In twenty-nine States sanitary organizations have been adopted, while eleven still refuse to make any provision for a State Board of Health, but when all the States have fallen into line then a majority of States will demand a National Board of Health.

Dr. Pratt paid a high tribute to the worth of the late Dr. Farr, of England, in originating the mode of collecting vital statistics. He gave descriptions of advanced methods of sanitary work in Michigan, and said we owe much of our advancement to ladies.

Paper was referred to the committee on publication.

The report of the treasurer was then read, showing a balance of $903.96.

The Librarian reported an addition during the year of 112 distinct titles, making an aggregate of 5,713 distinct volumes, including monographs and reprints from library exchanges. The Librarian recommends that $200 be placed at the disposal of the library for securing and binding pamphlets, and $50 for the usual subscription to the Index Medicus. The reports accepted and recommendations adopted.

The Committee on Publication, Dr. Fricke chairman, announced that an index to the twenty-three volumes of the asssociation transactions was in course of preparation by the Librarian, of which 1,500 copies had been issued at a cost of $500, and would be sold to the members at $1 per volume. Report adopted.

After some delay the Committee on Nominations made the following report through the chairman, Dr. Eugene Grissom:

Place and time of meeting, Washington, first Tuesday in May, 1884.

*For President, next year*, AUSTIN FLINT, New York; *Vice President*, R. A. KINLOCH, Charleston, S. C.; *Second Vice President*, Dr. T. B. LESTER; *Third*, Dr. A. L. GIHON, U. S. N.; *Fourth*, DR. S. C. GORDON, Portland, Me.; *Treasurer*, R. J. DUNGLISON, Philadelphia; *Librarian*, DR. C. H. A. KLEINSCHMIDT, of Washington, D. C.; *Chairman of Committee of Arrangements*, DR. A. Y. P. GARNETT, of Washington, D. C.; *Assistant Secretary*, DR. D. W. PRENTISS.

The Judicial Council consists of Drs. F. D. Cunningham, Virginia; H. O. Marcy, Massachusetts; W. O. Baldwin, Alabama; J. S. Billings, U. S. A.; T. W. Miller, U. S. Marine Hospital Service; Eugene Grissom, North Carolina; R. N. Todd, Indiana; to fill vacancy, E. W. Clark, Iowa.

Chairmen of the sections were announced as follows:

Practice of Medicine, J. V. Shoemaker, of Pennsylvania; Obstetrics and Gynæcology, T. A. Reamy, of Ohio; Surgery and Anatomy, Chas. T. Parks, of Illinois; Ophthalmology, J. J. Chisholm, of Baltimore; Diseases of Children, Wm. Lee, of Indiana; State Medicine, J. D. Roberts, of Tennessee; Aural Surgery, T. W. Brophy, of Illinois.

The Committee on State Medicine is composed of one member from each State, the names of whom were read. Also the Committee on Necrology, Dr. J. M. Toner, chairman, whose names were also read.

Dr. Gihon presented a statement that the report originating in a careless interpretation of his address on the first day before the Section on State Medicine, that he was opposed to the Code of Ethics, was entirely untrue, and that he was a strong adherent of the present Code, and should continue to be at all times. Received with much applause.

Dr. Didama, of New York, then presented the following:

*My Dear Doctor :* Circumstances rendering it necessary for me to return early to-day to New York, will you kindly express to our brethren, the members of the American Medical Association, with my sincere thanks, an assurance that I thoroughly appreciate the great honor which has been conferred.

I accept the honor, feeling assured that I may confidently expect co-operation and indulgence in my efforts to fulfill the duties which it involves. Yours very truly,

AUSTIN FLINT.

The Association then adjourned to 9 o'clock Friday.

*Section on Practical Medicine* met in the Opera House, Dr. Hollister in the chair; Dr. Lee, Secretary.

Three papers were read: "Elements of Prognosis and Therapeutics in Laryngeal Tuberculosis," by J. Solis Cohen, of Pennsylvania, which was illustrated by enlarged plates showing the condition of the throat in laryngeal tuberculosis, and tubercular deposit in the epiglottis.

The prognosis of each case, the author said, lies in its own history, and is less grave in some cases than others. The course of laryngeal tuberculosis may be retarded more or less by treatment. In incipient cases, now and then, the development of the disease might be retarded sufficiently to start the patient toward health again.

Dr. H. A. Martin gave a long and elaborate history of "Vaccination and the Propagation of Vaccine Virus." He claimed the honor of being the sole and only originator of the propagation of virus in this country.

A fine volunteer paper, by Dr. T. A. Keyt, of Ohio, on "The Condition of the Arteries in Valvular Insufficiency," received the close attention of the section, and was briefly discussed and commended by Dr. Palmer, of Michigan, and others.

Section adjourned.

*Section on Gynæcology* met at Frohsinn Hall, 2:30 P. M., Dr. Bartlett in the chair; Dr. J. F. Jelks, Acting Secretary.

Dr. Battey, of Georgia, not present; his paper on "Battey's Operation, Death from Ether," not read.

Dr. P. Zenner, of Ohio, read a paper on "Value of Gynæcological Treatment in Hysteria and Allied Affections." The author took ground that Gynæcological measures were too frequently resorted to in nervous diseases, and that it is time to recognize that such measures may do harm as well as good. Many cases occur where the cure of the uterine disease does not affect the nervous malady; also uterine disease may remain while the nervous affection is cured. However, the same soil favors development of both. The author strongly condemned the promiscuous examination of maidens or young married women, mainly because there are nervous symptoms. General treatment is of the utmost value in such cases. The hygienic education should be attended to from childhood up to womanhood in predisposed females, and the physician should point out to parents a system of education that will soundly develop both body and mind, and lead to habits of self-control and unselfishness, most especially at the period of puberty, by suggesting useful employment or earnest study. He should guard against means that heat a naturally fervid imagination, and above all endeavor to keep the mind from the genital function.

Dr. G. M. Maughs, of Missouri, read an interesting paper on the gynæcology and midwifery of the ancients—a curious and interesting paper, which demonstrated that many of the rather advanced ideas of the present day were familiar to the physicians of the first century.

*The Section on Ophthalmology, Otology and Laryngology* met at the Board of Education chamber at 2:30 P. M., Dr. Chisholm in the chair. Dr. Carl Seiler, secretary.

Dr. Rumbold, of St. Louis, read a paper on the "Appearance of the Diseased Mucous Membrane of the Nose and Throat of

Adult Patients." Said the subject was too large for discussion, but his address would consist of remarks and hints upon the subject taken from an average history of an hundred cases—that the appearance of the pharynx of smokers was characteristic, and detailed the peculiar types and their probable prognosis as to length of treatment. He remarked it was not safe to take the patients' account of his symptoms. That Follicular inflammation does not need treatment, particularly as none of the symptoms are usually removed with them. Pharyngitis and laryngeal aphonia are frequently dependent upon irritation from inflamed nasal cavities, and are relieved accordingly.

In the discussion which followed, Dr. Seiler agreed partly in that there may be cases of aphonia relieved as above; the appearance of the pharynx he thought no indication of length of treatment required; did not agree with the author's description of inflamed throat; they usually vary more. He thought aphonia from laryngitis most frequent about puberty as hysterical paralysis, or about the time of change of voice in boys. He also disagreed as to the appearance of chronic smokers' throats. Thought cigarettes more irritating to the mucous membranes than the pipe or cigar; did not agree as to the harmlessness of the inflamed follicles; touching one of them will produce symptoms of violent congestion.

Dr. Rumbold remarked that probably not ten per cent. of the smokers in the room had white vocal cords.

A paper followed by Dr. J. J. Chisholm, entitled "Is Abscision a Proper Operation?" The paper considered the best mode of forming the stump for the artificial eye. Said that the stump left from abscision was extremely liable to ciliary irritation from the inclusion of the ends of the ciliary nerves in the cicatrix. Eye shells on stumps press on the whole length of the cicatrix, while in eyeless sockets they only impinge upon the tender surface at their periphery. Enucleation is one of the easiest of operations, abscision a most difficult one; injury to the ciliary body imminent and its immediate results incalculable.

Dr. Frothingham, of Michigan, agreed with the writer, and urged the abandonment of abscision; he also dilated upon the imminence of subsequent inflammatory action. He mentioned the difficulty of retaining observation of the patients, who would frequently fail to return until in a hopeless condition. The stump was also a constant source of danger from deposit or morbid growth, and its retention was only a cosmetic effect after all.

Dr. Lundy, of Detroit, also agreed as to the experience. There was possibly a little better motion to the artificial eye, but the risks far overbalanced this slight advantage. He gave an instance of bone deposit in the ciliary body; also gave cases of sympathetic ophthalmia, in which hopeless blindness had followed abcision.

Dr. Thompson, of Indiana, mentioned the necessity for extreme care in rounding off the stump in abscision, so that no slight projection should be left to irritate. He had also had cases of pan-opthalmitis following abscision, and had enucleated the eye in patients who had returned to light business on the second day.

Dr. Culbertson, of Ohio, was also in favor of the paper because of its thoroughness. He had never had an unfavorable result after abcision in thirty years' practice; did not know exactly why this was, but he never takes any stitches after abcision; leaves the eye then alone; the lens he allows to take care of itself. He had sometimes taken out the choroid and retina, and allowed the eye to take care of itself; this process results in not quite so large a stump, but, for practical purposes, one more useful. He thinks it a good plan to divide the tissues well back, as most of the sensitive parts are cut off.

The course of the discussion was still further in favor of enucleation and the abolition of abcision. The general opinion favored the axiom that a lost eye is a source of danger, and should be removed.

Dr. Cornwell, of Ohio, had removed the eye in several cases, together with the lachrymal gland, then stitched the ciliary margins of the lids together, closing the eye permanently, leaving drainage thread in the cavity till the small suppuration ceased.

The third paper by Dr. H. Culbertson, of Ohio, "A Form of Spectacles in lieu of Nose Pieces," demonstrated the use of extra semi-circular glasses, to be placed in a small frame in front and below the regular spectacles, to assist near vision. He claimed it was easy to prescribe them, and gave cases of astigmatic asthenopia and myopic astigmatism, which had been benefited by these glasses. Considerable skepticism as to the practicability of these glasses was manifested in the subsequent discussion, and it was generally esteemed unfortunate that the doctor should have lost the glasses after coming to Cleveland on the previous day.

Dr. Cornwell then read a volunteer paper detailing four interesting cases.

Dr. Seiler then presented a case of instruments in convenient shape for sudden night calls.

Dr. Turnbull read a letter from a patient concerning whose case his essay of the previous day was written.

Dr. A. C. Scott then read a letter from Dr. Calhoun, explaining his enforced absence as due to the severe illness of his wife.

Dr. Seiler then read the report of the committee on the publication of papers, the substance of which was that after careful consideration of the plan detailed in Vol. XXXIII. of the Transactions of the Association they had decided to recommend for publication only those papers read before this section by their authors.

Section then adjourned.

*Section on Diseases of Children.*—Met in council chamber at 2:30 p. m.

Interesting papers were read by Dr. H. N. Good, of Indiana, on "Dentition," which process he said was not a disease, but the affections accompanying it are many, from its irritation. Different susceptibility is a great factor in these affections. Reflex nervous irritability results in impaired digestion, in the evacuations from which are to be found myriads of bacteria, thus clearly tracing the cause of cholera-infantum. The thermal ranges are variously from 100 to 105°, pulse ranging in proportion. The author gave anodynes for the nervous disturbance and for the indigestion pepsin and bismuth. In the discussion following nearly all endorsed the opinions of the essayist. Dr. Reed, of Ohio, would not endorse the bacteria theory, and claimed the nervous irritation caused the diarrhœa, and could be arrested by lancing the gums and confining the diet to milk.

Then followed a paper from Dr. J. B. Casebeer, of Indiana, which was transferred from the first day, entitled "Pædiatric Medication and its Relation to General Medicine." Proper dosing he esteemed the main consideration in infantile therapeutics—more science in fixing the dose than in prescribing the medicine. The dose should be as palatable as possible, to be taken without perturbation. Some discussion followed the paper, by Dr. Sinnot, of Ohio, and Dr. Ulrich, of Pennsylvania, who declaimed against the excessive dressing and feeding of the new born. Dr. Von Kline, of Ohio, also made remarks.

The last paper, on "Infantile Paralysis," by Dr. Teale, of Indiana, was volunteered, and quite interesting. The substance of the paper was that infantile paralysis was too often pronounced incurable and should be given more attention. Dr. Snow, of Michigan, said, in the discussion which followed, that there was danger to children in the excessive use of the bromides, and had seen paralysis following their use.

After further discussion of the relations of croup and diphtheria, resumed from the previous day, the section adjourned.

*Section on State Medicine.*—Met and gave the entire afternoon to discussion of the resolutions offered on Tuesday by Dr. A. L. Gihon, U. S. N., at the close of his address. Before moving the adoption of his resolutions Dr. Gihon explained his position toward the Code. He stated that he had signed it, believed in it, and should stand by it; was opposed to anything which came in conflict with it.

The resolutions, which provided for the dropping from the list of the profession of all unqualified and illiterate members, and the impropriety of association with such, were finally laid on the table, after much argument pro and con.

Dr. Billings seemed to voice the opinion of the majority, when he said "that there was no necessity for these resolutions; that the Code says enough upon the subject, and that the general public is apt to jump to the conclusion that action of this kind is taken for the protection of those in the profession, to prevent other men from getting the fees. Action defining the qualifications necessary for physicians, organizing State Boards of Examiners should come from the people."

Dr. A. N Bell then offered a resolution urging upon the Association the importance of competent medical and sanitary service on ocean steamers.

Section adjourned *sine die.*

*Surgical Section* met at 2:30 P. M. in the Opera House, for the purpose of witnessing the "Illustration of Anatomical and Pathological Papers" by Dr. Alfred F. Holt, of Massachusetts, by the screen and oxy-hydrogen lantern. The exhibition was interesting, showing different pathological tissues and sections of the nervous system for inspection.

The section then repaired to Case Hall, where Dr. Verity, of Illinois, exhibited a convenient derrick for elevation of the subject in the application of the plaster jacket in spinal caries; also a complicated universal splint for suspension.

Dr. Wm. A. Byrd read a paper entitled "Excision of Both Hip Joints for Morbus Coxarious," and detailed an interesting case, in which the subject can now walk and attend school.

Dr. H. O. Marcy read a paper on "Surgical Treatment of Intestinal Obstructions," after which a discussion as to the use of the carbolic spray again arose. Dr. Gordon, of Maine, predicted that in a few years, physicians would be held criminally liable for the use of the carbolic spray.

Dr. Moon, of Kentucky, also opposed it.

Dr. Prewitt, of St. Louis, read a paper entitled "A Radical Cure for Ranula," in which he removes a section of the wall of the sac, causing its obliteration.

A paper on "The Early Use of the Trephine in Bone Affections," read by Dr. Ranzohoff' of Ohio, attracted close attention, and met the approbation of several gentlemen who discussed the paper briefly. Dr. Gunn, of Illinois, had practiced the author's plan twenty-five years.

Dr. Reynolds, of Michigan, then read a paper on "Urethral Stricture," in which there was nothing particularly new.

Dr. J. H. Warren, of Massachusetts, read an interesting paper on the subject of "Tissue Repair, or Pathology of Subcutaneous Injection for the Cure of Hernia."

Two papers, one from Dr. C. H. Wilson, on "A Form of Inguinal Hernia Liable to be Overlooked," and one from Dr. C. F. Gay, on "Syphilitic Mammary Tumors," referred to the committee on publication.

Section then adjourned.

Dr. Peck was obliged to leave before adjournment, and the chair was filled by Dr. A. B. Watson, of New Jersey, to the close.

### GENERAL SESSION—FOURTH DAY.

In Case Hall, 9:30 A. M. Friday. The attendance was perceptibly lighter, many members leaving on morning trains; quite a large number of ladies present.

Session opened with prayer by the Rev. Chas. Terry Collins, of Plymouth Church.

Dr. A. C. Miller, in the absence of Dr. X. C. Scott, Chairman of Committee of Arrangements, then announced the excursion in the afternoon to the country-seat of Mr. D. P. Eels—River Bank.

Dr. W. S. Smith, of Dakota, offered an amendment to the Constitution, providing for the admission of two delegates from the U. S. Indian Service, to be nominated by the Surgeon-General. Tabled.

Another amendment was introduced by Dr. Toner, of Washington, to the effect that the office of Permanent Secretary be abolished, and a secretary be nominated annually, who will serve without compensation, but was withdrawn in view of the change made in the duties of the Secretary, which enables him to serve without an honorarium.

An amendment offered by Dr. Sears, of Arkansas, toward the appointment of additional workers in the several sections, at the discretion of the chairmen and secretaries of sections, and that the Librarian be made a permanent officer, tabled one year.

Dr. Davis, in behalf of the Judicial Council, announced that the petition of Dr. D. W. Joy would be returned to him, with leave to supplement said paper with a written statement of the character of the new evidence he wished to introduce, and that the Council declines to act until the opening of the session of next year, from the impossibility of notifying all the parties concerned.

Also, in the case of Dr. Goodwillie, of New York, that the Council decided that his registration be canceled, and his dues returned to him. This action taken on account of Dr. Goodwillie's connection with the New York State Medical Society, which is heretical in its adherence to a code different from that of this Association.

Dr. Pratt, of Michigan, introduced the resolution of Dr. A. N. Bell, from the Section on State Medicine, upon the subject of competent medical and sanitary service on board all our trans-oceanic vessels, and a committee on the subject, consisting of Drs. A. N. Bell, A. L. Gihon, J. N. Quimby and H. H. Smith, were instructed to procure legislation on the subject, and report at the next meeting.

Dr. A. N. Bell then introduced a motion, in view of the fact that a number of papers had been read out of their proper sections, that hereafter all papers, except the President's address and those of the Chairmen of Sections, should be presented to the Trustees of the journal for assignment to their proper sections.

Dr. Toner wished to amend the motion by referring the papers to the Committee of Arrangements instead.

Dr. Pratt thought many papers were presented which should never have seen daylight at all.

Dr. J. Solis Cohen opposed the resolution, on account of having lost a valuable paper some years since through sending it to the committee. The motion was tabled by vote.

Dr. Turnbull, of Philadelphia, offered a resolution that the respective State Legislatures be petitioned to pass laws requiring all railroad employés, before taking charge of trains, to be examined for hearing. Referred to the Section on Otology, to be laid over a year under the rules.

Drs. W. Brodie and H. T. Walker were appointed delegates to the Canadian Medical Association.

Dr. Keller offered the following: *Resolved*, That as in the near future cremation will become a sanitary necessity in large cities and populous districts of the country, that the question be referred to the section on Hygiene; carried.

Dr. Brodie offered resolutions of respect and appreciation of the life and labors of the late Surgeon General, J. K. Barnes. Dr. Blount, Chairman of the Section on diseases of children, who was obliged to leave early in the day, was allowed to submit his address without reading. Dr. Toner moved the thanks of the Association to the Secretary and Treasurer for the efficient manner in which they had performed their duties.

Dr. Quimby offered a resolution of thanks to the profession and citizens of Cleveland for their cordial and bountiful hospitality.

Dr. Garcelon, of Maine, offered the following: *Resolved*, That the thanks of the American Medical Association be extended to the retiring President, Dr. J. L. Atlee, for the able, dignified and satisfactory manner in which he has presided over the deliberations of the assembly, and that he retires with the best wishes of every member of this Association for a long continuance of a life so highly useful, not only to the present, but to all future generations.

The retiring President then invited the Vice-President elect upon the stage, and regretting the absence of the President elect, whom he eulogised as the Laennec of America, introduced the second Vice-President elect, Dr. Lester, of Kansas City, who took the chair and declared the Association adjourned to meet in Washington, D. C., on the first Tuesday in May, 1884.

At 2.45 p. m. the members of the Association and their ladies repaired to the N. Y. C. & St. L. Depot, where a comfortable train awaited their pleasure, and responded to the invitation of Mr. and Mrs. D. P. Eels to visit their elegant summer residence, River Bank, situated six miles west of the city in a beautiful grove on a bluff overlooking Lake Erie. Mr. and Mrs. Eels received their guests, assisted by Dr. and Mrs. G. C. E. Weber and a few friends. Two pleasant hours were spent in social converse on the cool verandas or strolling about the grounds, enjoying the delightful contrast to the close confinement of the previous three days. The same train bore them back to the city, and by 9 p. m. very few members of the Association remained in Cleveland. The members were, without exception, highly pleased with the conduct and deportment of the meeting, unflagging interest being manifested from first to last. The attitude of the body with regard to the Code being dignified and unflinching, a disposition instantly manifest to resent any interference with any of its tenets in the least degree.

On Tuesday, one of the Ohio members, Dr. J. C. Hubbard, was fatally seized with apoplexy while enjoying the hospitality of Dr. G. C. E. Weber. The gentlemen were enjoying a cigar in the drawing room after dinner, when Dr. Hubbard suddenly sank to the floor and ceased to breath. Everything possible was done for the unfortunate doctor, but his heart ceased to beat in a few minutes. Dr. Hubbard lived in Ashtabula and was one of the most prominent physicians in Northern Ohio. He was sixty-four years of age.

On Tuesday night the profession of Cleveland entertained its visitors in superb style at the Euclid Avenue Opera House. The parquette being floored over from the stage to the dress circle, furnishing a commodious promenade. Here all were made welcome, the polished city Galen in his dress suit equally with the bucolic Esculapius in his less bending dignity.

Wednesday and Thursday evening a number of Cleveland's wealthiest citizens received the visitors at their homes and a detachment of the Profession of Cleveland were at each residence to introduce the strangers to their hosts.

